# Label-free three-dimensional imaging of *Caenorhabditis elegans* with visible optical coherence microscopy

**DOI:** 10.1371/journal.pone.0181676

**Published:** 2017-07-20

**Authors:** Séverine Coquoz, Paul J. Marchand, Arno Bouwens, Laurent Mouchiroud, Vincenzo Sorrentino, Daniel Szlag, Johan Auwerx, Theo Lasser

**Affiliations:** 1 Laboratoire d’Optique Biomédicale, École Polytechnique Fédérale de Lausanne, Lausanne, Switzerland; 2 Laboratory of Integrative and Systems Physiology, École Polytechnique Fédérale de Lausanne, Lausanne, Switzerland; Universitat Zurich, SWITZERLAND

## Abstract

Fast, label-free, high-resolution, three-dimensional imaging platforms are crucial for high-throughput *in vivo* time-lapse studies of the anatomy of *Caenorhabditis elegans*, one of the most commonly used model organisms in biomedical research. Despite the needs, methods combining all these characteristics have been lacking. Here, we present label-free imaging of live *Caenorhabditis elegans* with three-dimensional sub-micrometer resolution using visible optical coherence microscopy (visOCM). visOCM is a versatile optical imaging method which we introduced recently for tomography of cell cultures and tissue samples. Our method is based on Fourier domain optical coherence tomography, an interferometric technique that provides three-dimensional images with high sensitivity, high acquisition rate and micrometer-scale resolution. By operating in the visible wavelength range and using a high NA objective, visOCM attains lateral and axial resolutions below 1 μm. Additionally, we use a Bessel illumination offering an extended depth of field of approximately 40 μm. We demonstrate that visOCM’s imaging properties allow rapid imaging of full sized living *Caenorhabditis elegans* down to the sub-cellular level. Our system opens the door to many applications such as the study of phenotypic changes related to developmental or ageing processes.

## Introduction

Imaging of biological processes *in vivo* in a whole organism is of major interest for biology and life sciences. This is of particular importance for studies of complex phenomena such as morphogenesis or ageing. The nematode *Caenorhabditis elegans* (*C. elegans*) is a well studied model organism for understanding these processes and has been used in many research projects in biology. Its widespread use is mainly due to its small size, rapid life cycle, ease of culturing in laboratory environments, transparency, and fully sequenced genome.

Current gold standards for optical imaging of living *C. elegans* are differential interference contrast (DIC) and confocal fluorescence microscopy. These two techniques mainly provide sub-micrometer two-dimensional images of cellular and sub-cellular structures. However, DIC is not capable of tomographic imaging as it does not provide optical sectioning. Confocal microscopy allows volumetric imaging but with limited speed due to the required three-dimensional (3D) raster scan. Additionally, fluorescence microscopy requires labels which can interfere with cellular processes. Over the past decades, several technologies such as spinning-disk, light-sheet or two-photon microscopy have emerged to improve the acquisition speed, to increase the penetration depth and/or to reduce photobleaching and phototoxicity [1–8]. These techniques however still rely on labeling, potentially interfering with the native cell behavior.

Alternative concepts for 3D imaging based on the intrinsic optical properties of the sample exist. Among them, optical projection tomography (OPT) has been shown to be a powerful tool for the visualization of specimens ranging between 1 to 10 mm with a 3D resolution of a few micrometers [9–16]. OPT has the advantage of using both absorption and fluorescence as source of contrast. A microscopic OPT platform especially suited for *in vivo* imaging of *C. elegans* was presented in [14]. Even though this OPT system provides an exceptionally high 3D resolution of about 2 μm, its resolution is still outperformed by about 4-5× by classical fluorescence microscopy and is at the limit for cellular resolution. Another interesting label-free method which has been applied to 3D visualization of *C. elegans* is tomographic phase microscopy [17]. This technique provides 3D refractive index maps of cells and multicellular organisms with a lateral resolution of 0.5 μm and an axial resolution of 0.75 μm approximately. The image of a worm acquired with this method showed several well visible internal structures such as the pharynx and digestive tract. However, the drawback is that due to the limited depth-of-field (DOF), the objective focus has to be scanned over intervals of ∼15 μm for accurate reconstruction when measuring samples with extended thickness. Recently, a novel lens-free optical tomographic microscope has been introduced combining simultaneously a wide field-of-view of ∼15 mm^2^ and a long DOF of ∼1 mm with a lateral resolution of <1 μm and an axial resolution of <3 μm [18]. Beside the large volume probed, the compact, lensless and almost alignment-free scheme could make this system a powerful tool for high-throughput imaging and screening. As a demonstration, a *C. elegans* is imaged, showing distinct details in different parts of the nematode. This technique comes, however, with several limitations such as a lower than diffraction-limited resolution and imaging artifacts well described in [18].

To provide an alternative which addresses the challenges mentioned above, we demonstrate *in vivo* imaging of whole *C. elegans* down to the sub-cellular level with visible optical coherence microscopy (visOCM), a fast, label-free, and highly sensitive optical system with 3D sub-micrometer resolution which we introduced recently [19]. Our setup is derived from optical coherence tomography (OCT), an interferometric technique that provides 3D images with micrometer resolution of biological samples [20–23]. OCM is based on the same principles as OCT but uses optics with higher numerical aperture (NA), increasing lateral resolution. The image contrast in OCT and OCM results from variations of index of refraction which are strongly amplified due to the underlying interferometric imaging. Unlike confocal microscopy, the axial resolution in OCT/OCM is determined by the spectral width of the light source (Δλ). The lateral resolution is dependent on the NA of the objective, creating a trade-off between transverse resolution and DOF in a classical optical setting. To circumvent this limitation, we have replaced the traditionally used Gaussian beam by a Bessel beam generating an elongated illumination field [24]. This technique, termed extended-focus OCM (xfOCM), has been applied to *in vivo* imaging of islets of Langerhans and cerebral β-amyloid plaques in mice [25–29]. Moreover, the detection in the Fourier domain offers high sensitivity and fast acquisition rates [30]. The combination of all these characteristics makes xfOCM a powerful tool for rapid 3D *in vivo* imaging. So far, xfOCM systems employ a near-infrared broadband light source centered at λ_0_ = 800 nm and a 10×/0.3NA objective providing an axial resolution of 2 μm and a lateral resolution of 1.3 μm which is maintained over a depth of 400 μm [25–29]. For imaging *C. elegans* whose diameter typically ranges from 50 to 80 μm in adulthood, such a deep penetration is not required. The depth of field can therefore be traded against a higher transverse resolution by using higher-NA optics. Furthermore, the transparency of *C. elegans* invites for imaging in the visible wavelength range with a further gain in resolution. Using a broadband spectrum in the visible wavelength range not only improves the diffraction-limited lateral resolution but also generates a sub-micrometer axial resolution, given by the coherence length proportional to λ_0_^2^/Δλ. Altogether, these unique characteristics of our platform enable rapid label-free 3D imaging, demonstrated here by high contrast, high accuracy and high resolution images of living *C. elegans*.

## Materials and methods

### visOCM setup

**Fig 1:**
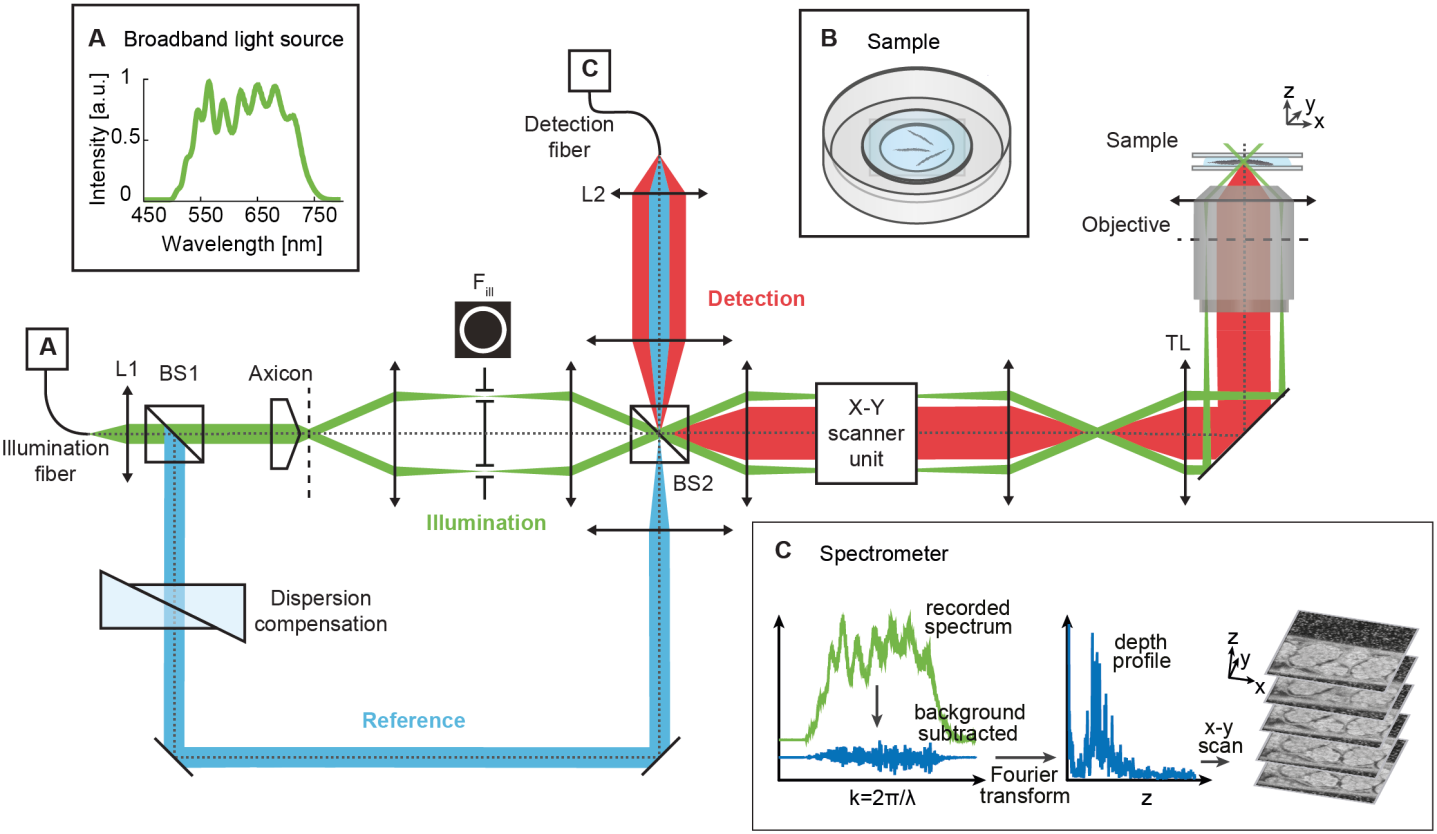
Schematic of the visOCM setup for *C. elegans* imaging. Light from a laser source with a broad spectrum in the visible range (**A**, inset) is collimated by lens L1 and split by beam-splitter BS1 into a sample (green) and reference (blue) arm. In the sample arm, the axicon lens generates a Bessel-like illumination beam which is then guided to the tube lens (TL) and objective by the X-Y galvo-scanner unit. The back-reflected light (red) from the sample (**B**, inset) is recombined with the reference arm by beam-splitter BS2 and focused by L2 into the detection fiber. Finally, the spectrometer (**C**, inset), records the interference pattern which is processed to yield a depth profile of the *C. elegans* structure. The data processing steps are illustrated in S1 Fig. Scale bars: 25 μm.

The core of our visOCM setup is a Mach-Zehnder interferometer with decoupled illumination and detection paths [25, 31]. The broadband spectrum from a supercontinuum source (Koheras SuperK, NKT Photonics) is bandwidth-limited to a wavelength range of Δλ ≃ 160 nm centered at λ_0_ = 590 nm, resulting in an axial resolution of ∼0.72 μm in water. This light is injected in the interferometer and split into reference and sample beams by a first beam-splitter (BS1 in Fig 1). An axicon lens (Del Mar, 175°) and a telescope in the sample arm generate a Bessel illumination. The telescope contains an annular mask F_ill_ to block stray light from imperfections of the axicon tip. In the reference arm, prism pairs of different glasses (BK7, SF10 and UVFS) are placed for matching the dispersion in both arms. The illumination beam is then raster scanned in the lateral dimensions by galvanometric scanners (Cambridge Technology). A 40× water immersion objective (Olympus, NA = 1.15, effective NA = 0.86) mounted in an inverted configuration illuminates the sample. The high NA objective and the Bessel beam provide a high lateral resolution of ∼0.5 μm in water uniformly maintained over an extended focus of ∼40 μm. The back-scattered light is then collected, superimposed to the reference beam by a second beam-splitter (BS2), and recorded by a custom-designed spectrometer consisting of a transmission grating (600 lines/mm) and a line scan camera (spL2048-140km, Basler Sprint) providing an axial scan (A-scan) rate of up to 140 kHz. For more technical details on the visOCM setup, we refer to [19].

### Data acquisition and processing

In our visOCM setup, the illumination beam is raster scanned over the sample typically over a 100 × 100 μm^2^ area. The interferometric signal is acquired at a rate of 20 kHz (43 μs integration time) at 512 × 512 lateral positions. *C. elegans* worms prepared as explained in the next section are aligned along a scan axis. Several 3D image stacks are acquired to cover their full length (∼1 mm) by translating laterally the dish with two motorized scanning axes (Z606, Thorlabs). These images, overlapping by 10%, are then stitched together into a mosaic as described below. The illumination power on the sample is ∼3.5 mW. This power is distributed over the extended DOF and is scanned over the sample, thereby limiting light exposure. With these parameters, a high resolution 3D image of 512 × 512 × 2048 pixels corresponding to a volume of 100 × 100 × 500 μm^3^ is acquired in 13 seconds. A set of approximately 16 3D stacks is sufficient to image the entire *C. elegans* with sub-micrometer resolution. The acquisition time for one image stack could be significantly improved to <2 seconds for the same image size, by taking advantage of the full speed of our line detector (i.e. 140 kHz). This shorter integration time would however come at the cost of lower signal-to-noise ratio (SNR).

The signal processing steps leading to the final volumetric image of *C. elegans* are illustrated in S1 Fig. In OCM, a 3D image is a sequence of B-scans. In analogy to ultrasound, these B-scans are themselves composed of individual A-scans (depth profiles). At each lateral position, the recorded spectrum is processed to yield a depth profile, consisting in a background subtraction and a wavelength to wavenumber mapping followed by a Fourier transformation (Fig 1 inset C and S1 Fig). The depth structure is obtained by taking the logarithmic squared norm of this data. The image stacks are then re-scaled along the axial direction using an estimated index of refraction of n = 1.33 to represent geometrical rather than optical distance. The resulting images are finally assembled into a mosaic using the algorithm for stitching of tiled 3D microscopic image acquisitions described in [32] and available in ImageJ. A 3D Gaussian filter with standard deviation σ = 1 pixel (∼ 0.2 μm) is applied to the obtained 3D cropped images of whole *C. elegans*, which typically consist of approximately 7000 × 512 × 300 pixels. For all images presented here, no temporal averaging was used. The 3D rendering visualizations below were produced with the Imaris software (Bitplane).

### Sample preparation

Wild-type Bristol N2 *C. elegans* animals were cultured at 20°C on nematode growth media agar plates seeded with *Escherichia coli* (*E. coli*) strain OP50 following standard protocols [33]. Prior to imaging, the worms were immobilized with 10 mM tetramisole solution (Sigma-Aldrich), transferred to a glass-bottom dish (MatTek Corporation) and covered with a cover slip (Fig 1, inset B). Small drops of water were added on the sides of the dish to prevent evaporative loss.

## Results

**Fig 2:**
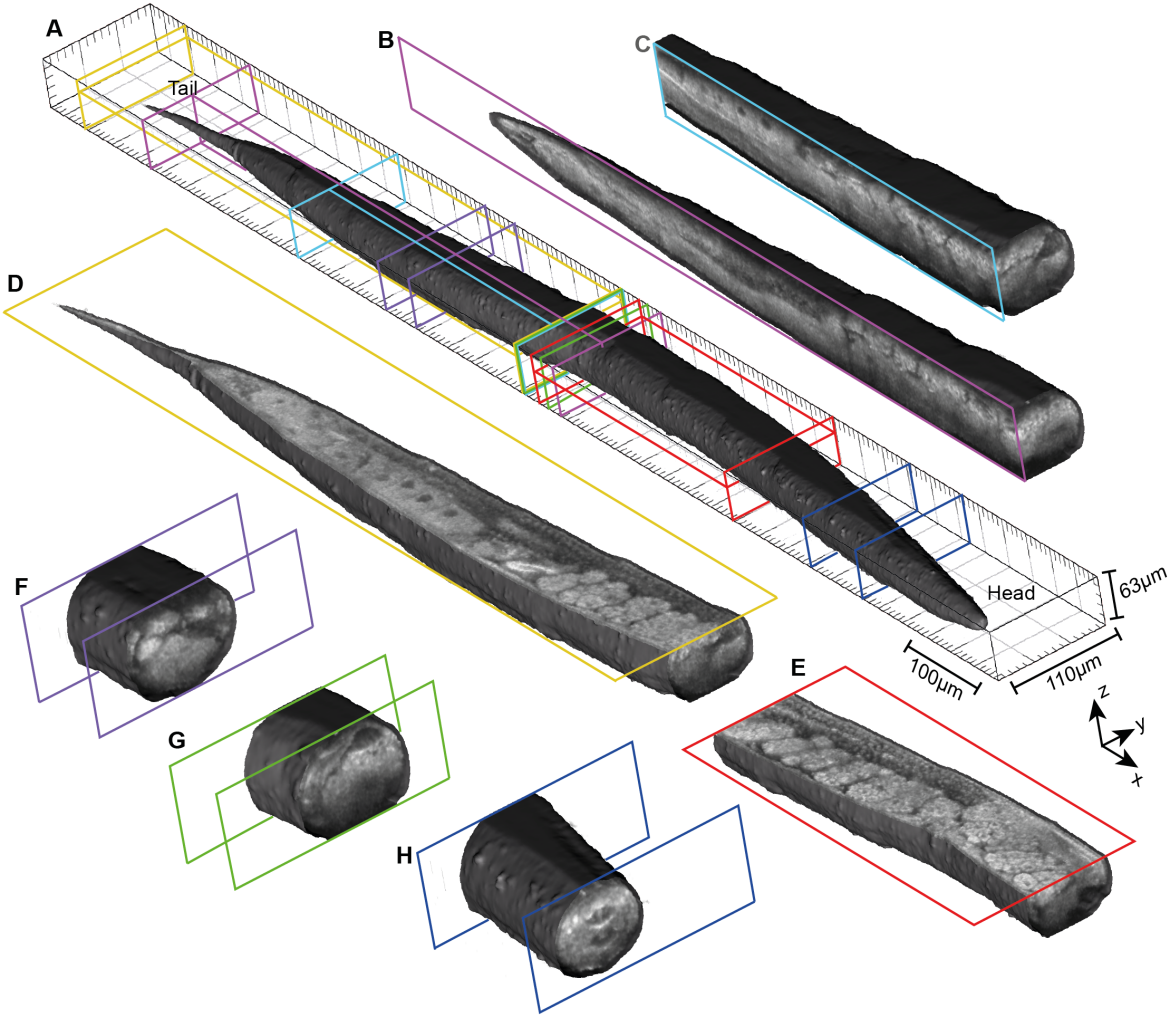
3D volume rendered images and cut sections of a young adult wild-type *C. elegans*. **(A)** 3D visualization of the body surface of the nematode. The location of the following cut sections are highlighted. **(B, C)** Sections along the y-axis. **(D, E)** *En face* (x-y) cuts. **(F-H)** Transverse sections. The high contrast and resolution of our visOCM setup reveal all major anatomical features of the worm. The reproductive tissue, including germ cells, oocytes and embryos, can be particularly well observed in these sections. See also S1 Video for an *en face* scan through the whole *C. elegans*.

**Fig 3:**
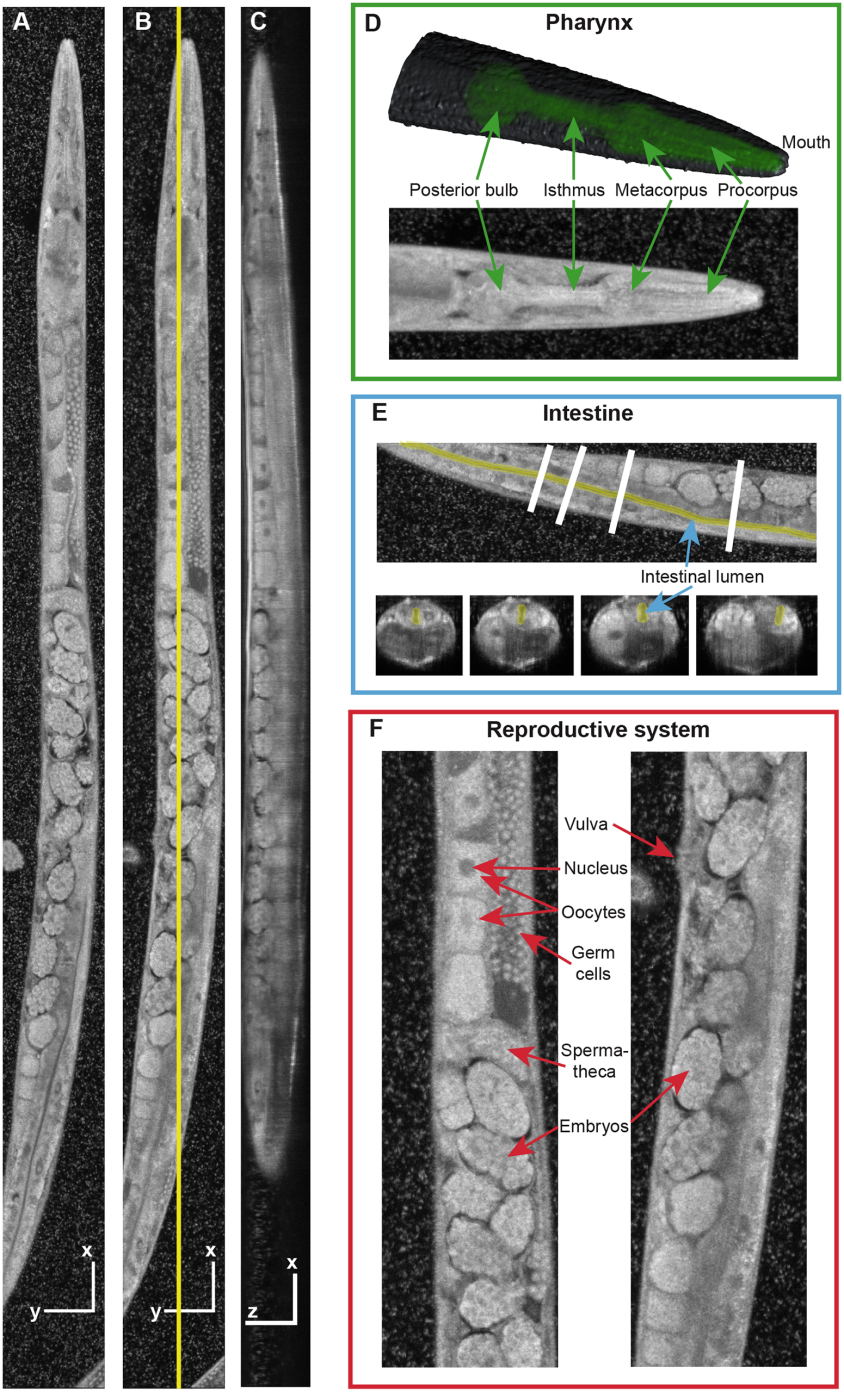
Anatomy of the *C. elegans* as revealed by visOCM. **(A, B)** *En face* projections at two different depths, and **(C)** side view at the location highlighted in **(B)**. Scale bars indicate 50 μm. **(D)** Top: A 3D rendered model of the head with the pharynx highlighted in green. Bottom: Maximum-intensity projection through the entire animal’s head. **(E)** *En face* view (top) and corresponding transverse sections (bottom), with the lumen of the intestine highlighted in yellow. **(F)** Zoom regions of the reproductive system showing germ cells, oocytes, spermatheca, embryos and the vulva. The 3D sub-micrometer resolution and the intrinsic contrast of our technique enable a clear and detailed visualization of tissue structures down to the sub-cellular level (see also S2 Video).

The performance of our system for rapid 3D sub-micrometer imaging of *C. elegans* anatomy is demonstrated in Figs 2 and 3. Fig 2 and S1 Video present a visOCM image of a wild-type fertile adult animal. The mosaic was assembled from 16 image stacks with a 100 × 110 μm^2^ optically scanned area, representing a total area of 1.26 mm × 110 μm. The performance of our method can be seen in Fig 2(B)–2(H) presenting cut sections along the three axes at the locations indicated in Fig 2(A). It can be seen that our technique reveals the 3D anatomy of the *C. elegans* with a high contrast, high resolution and high accuracy without labels or other contrast agents. This enables a clear identification of the different tissue structures of the worm. Furthermore, sub-cellular features such as nuclei are clearly resolved. The capacity of visOCM in terms of intrinsic contrast, sub-micrometer resolution and high sensitivity can be particularly well assessed by observing the reproductive system. Here, germ cells, oocytes, fertilized eggs and the egg-laying apparatus can be visualized in 3D with a very high level of details. Smaller anatomical features such as the rectum and the vulva are also visible (S1 Video).

Fig 3 presents the anatomy of *C. elegans* as imaged by visOCM in more details. As in Fig 2, the specimen under observation was a live young adult wild-type worm. The final volume shown in this figure is composed of 15 image stacks of 100 × 100 μm^2^ laterally. Fig 3(A) and 3(B) are *en face* (i.e., x-y) views at two different depths while Fig 3(C) shows a side view at the location indicated by the yellow line in Fig 3(B). Again, different anatomical features are clearly visible down to the sub-cellular level in the whole imaging volume. The small white spots around the *C. elegans* are *E. coli* bacteria transferred to the glass-bottom dish together with the worms. Fig 3(D)–3(F) focus on several *C. elegans* tissues. In Fig 3(D), a 3D rendering and a maximum-intensity projection of the head show that the pharynx and its different regions (corpus, isthmus, terminal bulb) are well distinguishable even without contrast agents. Fig 3(E) demonstrates that the lumen of the intestine can also be easily identified both in an *en face* view (top) and in transverse sections (bottom) along the animal, as highlighted in yellow. Finally, Fig 3(F) presents two zoomed-in areas of the reproductive system. All major parts (germ cells, oocytes, spermatheca, vulva, embryos) can be observed with high accuracy. The quality of the entire image is more readily appreciable in the 3D volume presented in S2 Video.

## Summary and discussion

In summary, we presented high-resolution *in vivo* label-free 3D imaging of *C. elegans* with visOCM, a recently introduced tomographic imaging method. The high NA objective, extended focus scheme and broadband visible light source enable 3D sub-micrometer imaging over a depth of ∼40 μm with high acquisition speed and high sensitivity inherent to OCM. Furthermore, no labeling or staining is required, thereby precluding issues such as photobleaching and phototoxicity.

To further extend the capabilities of our system, a 3D segmentation and analysis tool should be developed. This would allow to accurately quantify the volume of the different tissues and cells in the *C. elegans*, making visOCM a very interesting label-free, high-resolution, *in vivo* phenotypic platform. Moreover, in addition to the morphological information, other traits can also be assessed with our system. For example, the pharyngeal pumping rate, which undergoes a significant age-related decline, could be characterized with high precision by fast single B-scan imaging. With the A-scan rate used in this work, a B-scan or transverse view can be acquired at a rate of ∼40 Hz, approximately 8× faster than the maximum pharyngeal pumping rate occurring in young adults. Furthermore, as presented in [27, 28] for our previous xfOCM setup, a confocal fluorescence imaging channel could be added to our visOCM instrument to obtain molecular specificity along with the anatomical information.

Altogether, the images presented herein demonstrate that visOCM is a powerful method for fast and accurate visualization of *C. elegans* anatomy in 3D with high contrast down to the sub-cellular level, within the context of the whole organism. In comparison, 3D images of entire *C. elegans*, presented in [14, 17, 18] and acquired with other optical tomographic techniques, do not provide such a distinct, high resolution and detailed visualization of the structures of the nematode. In conclusion, we believe that the use of visOCM would greatly benefit studies of time-dependent, stochastic biological processes such as development, ageing or age-related diseases.

## Supporting information

S1 Fig Schematic of the data processing. The different steps are explained in the section dedicated to data acquisition and processing.
(TIF)

S1 Video visOCM *in vivo* imaging of a wild-type young adult *C. elegans*. The green inset shows a close-up of fertilized eggs and germ cells. Oocytes, intestine and rectum are visible in the cyan inset. An orthogonal view at the location highlighted by the white line is also shown (white rectangle).
(MOV)

S2 Video *En face* scan through the whole body of a *C. elegans* animal. The two insets highlighted in green and cyan show in more details the head of the nematode and the reproductive system including germ cells, oocytes, embryos and the spermatheca.
(MOV)
