# Supplementary figures and images for: Label-free three-dimensional imaging of Caenorhabditis elegans with visible optical coherence microscopy
Source: PLoS One. 2017 Jul 20;12(7):e0181676. doi: 10.1371/journal.pone.0181676 (PMC5519216; doi:10.1371/journal.pone.0181676)

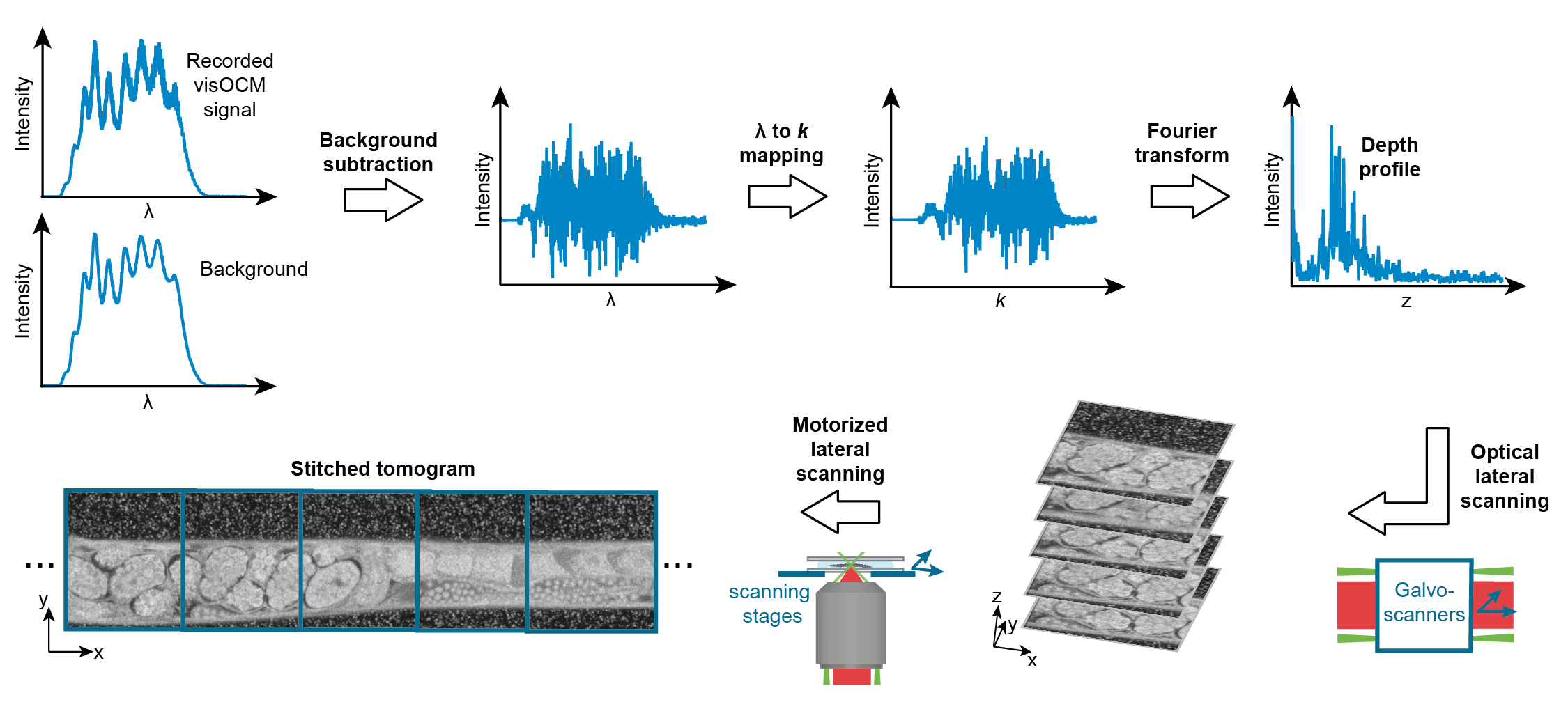

Supplement: S1 Fig — The different steps are explained in the section dedicated to data acquisition and processing. (TIF) [file pone.0181676.s001.tif]
